# Preclinical Pharmacokinetics of Scoparone, Geniposide and Rhein in an Herbal Medicine Using a Validated LC-MS/MS Method

**DOI:** 10.3390/molecules23102716

**Published:** 2018-10-22

**Authors:** Tun-Pin Hsueh, Tung-Hu Tsai

**Affiliations:** 1 Institute of Traditional Medicine, School of Medicine, National Yang-Ming University, Taipei 11221, Taiwan; melaopin@gmail.com; 2 Department of Chinese Medicine, Kaohsiung Chang Gung Memorial Hospital and Chang Gung University College of Medicine, Kaohsiung 83301, Taiwan; 3 Graduate Institute of Acupuncture Science, China Medical University, Taichung 404, Taiwan; 4 School of Pharmacy, College of Pharmacy, Kaohsiung Medical University, Kaohsiung 807, Taiwan; 5 Department of Chemical Engineering, National United University, Miaoli 36063, Taiwan

**Keywords:** Yin-Chen-Hao-Tang, tandem mass spectrometry, scoparone, geniposide, rhein, herbal medicine

## Abstract

The herbal formula Yin-Chen-Hao-Tang has been reported to have anti-fibrosis properties. The aim of this study was to reveal the pharmacokinetic characteristics of bioactive compounds in this herbal formula. A new high-performance liquid chromatography-tandem mass spectrometry method was developed and validated for simultaneous determination of scoparone, geniposide and rhein in rat plasma. A pharmaceutical herbal powder was administered to rats at doses of 1 g/kg and 3 g/kg orally. The method showed excellent linearity (r^2^ > 0.999) and validation was successfully conducted for the pharmacokinetic study. The results show that the C_max_ values and areas under the curve of scoparone, geniposide and rhein were higher and not proportional to the dose in rat plasma, while the T_max_ and half-life values were consistent in the group that received 1 g/kg. The clearance of the higher dose (3 g/kg) did not decrease proportionally to that of the low dose. The results showed the nonlinear pharmacokinetic properties of scoparone, geniposide and rhein in Yin-Chen-Hao-Tang that suggested possible accumulation of bioactive compounds through oral administration. This pharmacokinetic study reveals that an increased dose of this herbal formula would largely increase the maximum concentration and bioavailability of scoparone, geniposide and rhein.

## 1. Introduction

Chronic liver disease causes serious health problems globally due to the progressive dysfunction of the liver that leads to eventual fibrosis and cirrhosis. Viral hepatitis, alcoholic and non-alcoholic fatty liver diseases and autoimmune hepatitis are the most wide-spread forms of chronic liver disease that pathologically cause destruction and regeneration of the liver parenchyma, which subsequently activates the oncogenic pathways that promote liver tumor formation [1]. Advanced cirrhosis accounts for 39% of deaths world-wide and is a life-threatening condition with limited treatment options [2]. A high prevalence of chronic viral hepatitis infection related to hepatocellular carcinoma is evident in Eastern Asian countries, of which more than 50% of cases are HBV in Malaysia and Thailand and 27% of cases are HCV in Mongolia and Taiwan [3]. The actual ten-year survival rate for hepatocellular carcinoma is approximately 7.2 to 26.8%, depending on staging [4,5].

With the increasing prevalence of phytomedicines worldwide, traditional herbal medicines have been applied to alleviate several diseases not only for maintenance of health or prevention but also for improvement and treatment of physical or mental illness. *Artemisia capillaris* Thunb (Yin-Cen-Hao), *Gardenia jasminoides* Ellis (Zhi-Zi) and *Rheum officinale* Baill (Da-Huang) are used as a combined formula intended to address chronic hepatitis and jaundice. They were recorded in the ancient book “Shang-Han-Lun (Treatise on Cold Damage Diseases)” and formulated as Yin-Chen-Hao-Tang (YCHT, which means *Artemisia capillaris* decoction). The population diagnosed with chronic hepatitis and prescribed the herbal decoction Yin-Chen-Wu-Ling-San containing this formula was found to be 10.8% in Taiwan in 2002 [6]. The therapeutic effect of this formula demonstrated that it had anti-fibrosis and anti-apoptosis properties and alleviated oxidative stress in the liver when fed in vivo [7,8,9,10]. The major medicinal herb *Artemisia capillaris* Thunb in the formula was experimentally verified to have bioactive compounds, including β-sitosterol, quercetin, capillarin, capillarisin, scoparone (6,7-dimethylesculetin) and cirsimaritin [11]. Scoparone isolated from *A. capillaris* showed antioxidant properties in cold-preserved rat hepatocytes by reducing malondialdehyde (MDA) and alanine aminotransferase (ALT) levels [12]. The anti-inflammatory activities of scoparone were revealed to attenuate tumor necrosis factor (TNF)-alpha, interleukin (IL)-1beta and IL-6 and to suppress inducible nitric oxide synthase (iNOS) and cyclooxygenase-2 (COX-2) in IFN-gamma- or LPS-stimulated cells [13]. There is some evidence for scoparone as a potential hepatoprotective candidate for hepatitis therapy [14].

*Gardenia jasminoides* Ellis is another medicinal herb that also has anti-inflammatory, anti-angiogenic and choleretic effects through the YCHT formula [15,16,17]. The constituents of its extracts including gardening, chlorogenic acid, artemisetin and especially genipin and geniposide [11]. Geniposide and genipin have been discovered to act against hepatic ischemia with reperfusion injury by reducing oxidative stress and apoptosis [15]. Geniposide also has been revealed to have anti-inflammatory activities by blocking the downregulation of toll-like receptor 4 (TLR4) expression that is upregulated by lipopolysaccharide (LPS) [18]. The pharmacological effects of geniposide also exhibited inhibition of histamine when released on atopic dermatitis and protective effects against ischemia and reperfusion-induced renal injury [19,20].

The other medicinal herb in YCHT is *Rheum officinale* Baill. Radix et *Rhizoma Rhei* contains known constituents including aloeemodin, emodin, epicatechin, rhein and gallic acid. The bioactive compound rhein was found to have pharmacological effects including anti-inflammatory, anti-allergic and anticancer functions [21,22,23]. Rhein triggers apoptosis via ER-stress associated pathways in primary human hepatic HL-7702 cells [24]. Rhein was also found to modulate the related sonic hedgehog and serine-threonine kinase signaling pathways that consequently suppress the mRNA and protein levels of fibrotic and tumorigenic mediators, including type I collagen, fibronectin, *N*-cadherin and matrix metalloproteinases in mammalian cells [25]. The pharmacological mechanisms of the antitumor properties of rhein were discovered to be active in multiple pathways.

High-performance liquid chromatography (HPLC) coupled with tandem mass spectrometry (MS/MS) techniques has been used for the identification and quantitation of complex biological matrices, including a wide variety of manufactured and natural chemicals, due to its highly sensitive and selective detection. The current trend in pharmaceutical analysis using tandem mass spectrometry is not only a movement towards quality control by quantifying bioactive compounds levels in herbal medicines but also a movement towards applying developed methods for pharmacokinetic studies of medicinal herbs [26,27,28]. Multiple compositional medicinal herbs for treating chronic liver disease under clinical conditions should also be noted for their pharmacokinetic parameters for safe administration [29]. Previous pharmacokinetic studies of YCHT used HPLC to determine 6,7-dimethylesculetin and geniposide in rat plasma [30]. Additionally, investigating bioactive components of YCHT in rat urine by UPLC-ESI-MS has also been described [31]. Nevertheless, commonly applied pharmaceutical products of YCHT analyzed by highly sensitive UPLC-MS/MS with a quantitative internal standard and the dose relation has never been identified. This study aimed to simultaneously investigate the pharmacokinetic parameters of the bioactive compounds scoparone, geniposide and rhein in two doses of YCHT using UPLC-MS/MS, not only to provide an analytical reference for herbal medicines but also to provide efficacy and safe treatment data for clinical administration of these medicines to patients.

## 2. Results

### 2.1. LC-MS/MS Optimization Conditions

**Figure 1:**
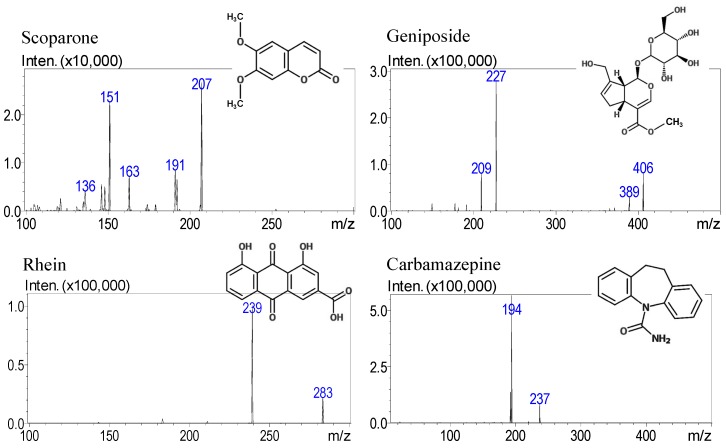
ESI-MS/MS spectra of bioactive constituents of the Yin-Chen-Hao-Tang formula and the internal standard. Scoparone (M + H)^+^, geniposide (M + NH_4_)^+^ and carbamazepine (M + H)^+^ spectra are obtained using ESI^+^ while rhein (M − H)^−^ was in ESI^−^.

Standard solutions of scoparone, geniposide, rhein and carbamazepine at 100 ng/mL were analyzed to optimize the mass spectrometry conditions. Full scans in positive and negative mode were investigated by monitoring both precursor and product ions in multiple reaction monitoring (MRM) mode to identify the maximum response of the analytes. The resulting highly selective and sensitive product ion and precursor ion spectra for the quantification assays of scoparone, geniposide and rhein are shown in Figure 1. The mass transition of the precursor-product ion was *m*/*z* 207 → 151 for scoparone, *m*/*z* 406 → 227 for geniposide, *m*/*z* 283 → 239 for rhein and *m*/*z* 237 → 194 for carbamazepine (IS) (Table 1). The observed *m*/*z* transition of geniposide was *m*/*z* 406 (M + NH_4_)^+^ instead *m*/*z* 389 (M + H)^+^ indicates an adduct as (M + NH_4_)^+^ due to the containing ammonium acetate in the mobile phase. The fragmentation pattern of target compounds in the study to determine the bioactive compounds in the pharmaceutical herbal product and the subsequent pharmacokinetic studies are compatible with previous reports for quantitative mass transitions of these analytes [26,32,33]. The optimization of the chromatographic condition to separate the analytes was conducted with respect to mobile phase composition, column and elution. The use of methanol and ammonium acetate as the mobile phase to achieve good sensitivity and a better peak shape was observed and the peak shape and signal intensity were improved by adding 0.1% formic acid. As a consequence, 1 mM ammonium acetate with 0.1% formic acid solution (gradient elution) was used for high-sample throughput.

### 2.2. Method Validation

#### 2.2.1. Specificity and Selectivity

**Figure 2:**
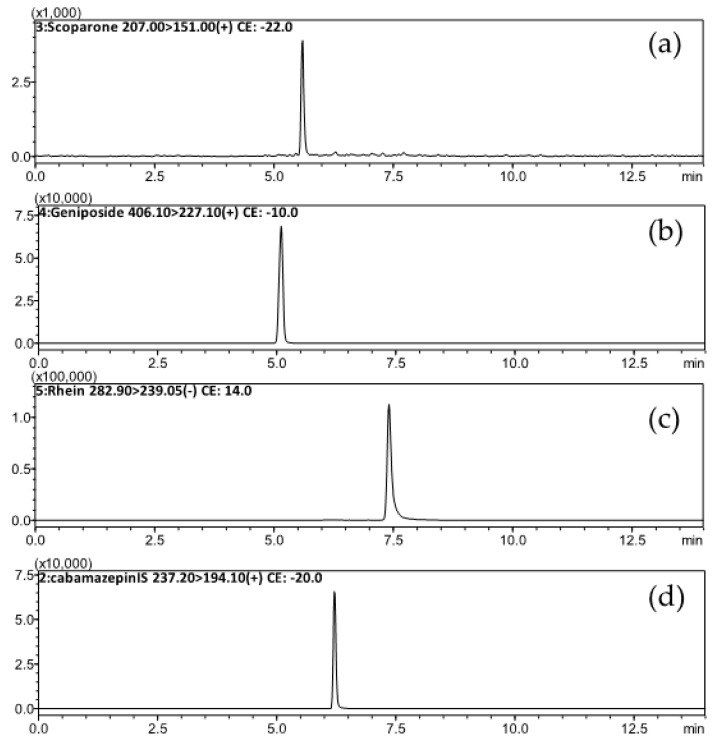
Typical MRM chromatograms of plasma samples collected 180 min after oral administration of the herbal formula Yin-Chen-Hao-Tang (3 g/kg): (**a**) scoparone (6.2 ng/mL) RT: 5.6 min, (**b**) geniposide (287.4 ng/mL) RT: 5.1 min, (**c**) rhein (350.3 ng/mL) RT: 7.4 min and (**d**) internal standard: Carbamazepine (10 ng/mL) RT: 6.2 min.

A sensitive and reliable analytical method was developed and validated under the optimized UPLC-MS/MS conditions to investigate the comparative pharmacokinetics of scoparone, geniposide and rhein in rats. Blank plasma samples using a protein precipitation procedure have a suitable recovery with the UPLC-MS/MS conditions to ensure less interference of the analytes and internal standard (IS) from plasma. The representative chromatograms for standards of scoparone, geniposide, rhein and IS spiked in blank rat plasma and for plasma containing these analytes collected at 180 min after administration of herbal products are shown in Figure 2. The results show no significant interference from endogenous substances observed under the current analytical conditions, which indicated the specificity and selectivity of the elaborated procedures.

#### 2.2.2. Linearity and the Limits of Detection and Quantification

The linearity of the calibration curves was determined and analyzed with six replicates of concentrations ranging from 5 to 1000 ng/mL in blank plasma samples. A calibration curve was plotted for each concentration, comparing the peak area ratio to the internal standard. The results demonstrated linearity of 5–500 ng/mL for scoparone and 10–1000 ng/mL for geniposide and rhein in rat plasma with correlation coefficients (r^2^) > 0.999 obtained for the regression lines. The limits of detection (LOD) and quantification (LLOQ) for all three bioactive components were 1 and 5 ng/mL, which showed excellent reproducibility and sufficient concentrations for oral administration of the herbal formulation in the subsequent PK study (Table 2).

#### 2.2.3. Precision and Accuracy

The intra-day and inter-day precision (% RSD) and accuracy (% bias) data for three bioactive components on five different QC samples are presented in Table 3. The intra-day precision and accuracy values for scoparone ranged from 0.1–7.9% and −0.2–5.6%, respectively, while those for geniposide ranged from 0.2–6.9% and −0.7–7.2%, respectively. Rhein had precision and accuracy values within 0.3–6.8% and −2.4–0.5%, respectively.

The inter-day precision and accuracy values were 1.7–4.1% and -3.7–2.0% for scoparone and 1.2–5.0% and −0.1–6.6% for geniposide, respectively. The inter-day values of precision and accuracy ranged between 1.6 to −3.1% and −3.0 to −1.1%, respectively, for rhein at five QC levels (Table 3). The precision and accuracy data were all within ±15% of that nominal values, indicating that the method was acceptable.

#### 2.2.4. Extraction Recovery and Matrix Effect

The extraction recovery of analytes from rat plasma was performed after the extraction procedure was assessed in three QC samples. The mean recoveries for scoparone and geniposide were 99.7 ± 7.2% and 91.9 ± 7.1%, respectively and those for rhein and IS were 109.5 ± 6.3% and 85.7 ± 5.2% in rat plasma, respectively. The matrix effect was 106.7 ± 6.7% for scoparone, 139.8 ± 17.2% for geniposide and 86.2 ± 4.7% for rhein. Table 4 contains the corresponding analytes in the analytical experiment. The calculated values had observed ion enhancement for geniposide and slight ion suppression for rhein but revealed consistent, reproducible and precise content.

### 2.3. Pharmacokinetics of Scoparone, Geniposide and Rhein in Rats

**Figure 3:**
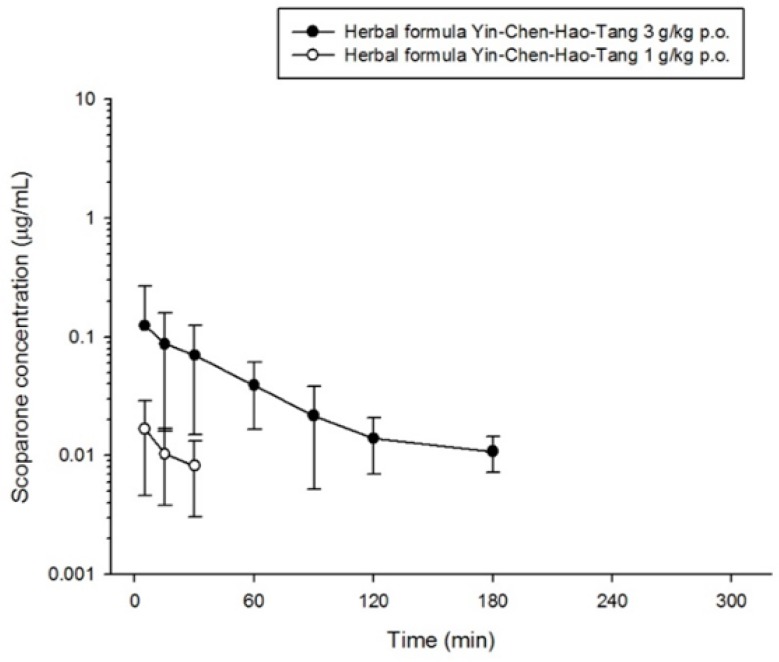
Concentration-time profile of scoparone in rat plasma following oral administration of different dose of herbal formula (1 g/kg p.o. or 3 g/kg p.o. Yin-Chen-Hao-Tang).

**Figure 4:**
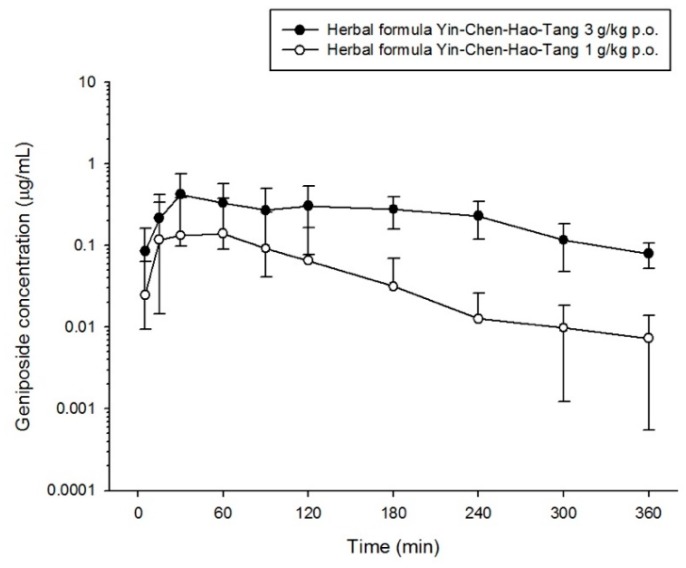
Concentration-time profile of geniposide in rat plasma following oral administration of different dose of herbal formula (1 g/kg p.o. or 3 g/kg p.o. Yin-Chen-Hao-Tang).

**Figure 5:**
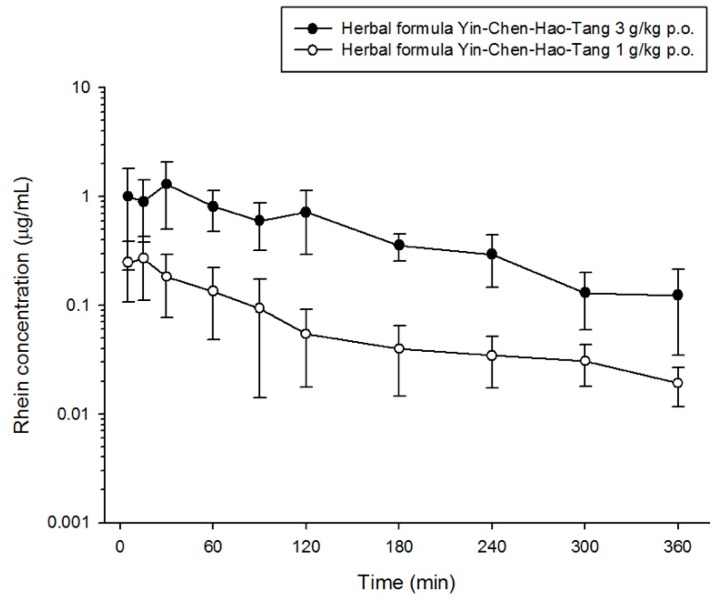
Concentration-time profile of rhein in rat plasma following oral administration of different dose of herbal formula (1 g/kg p.o. or 3 g/kg p.o. Yin-Chen-Hao-Tang).

The validated method was successfully applied to determine scoparone, geniposide and rhein in rat plasma by orally administering the herbal product YCHT at doses of 1 g/kg or 3 g/kg to rats. The mean plasma concentration-time profiles of each bioactive component in the herbal formulation are shown in Figure 3, Figure 4 and Figure 5. The maximum concentration (C_max_) of scoparone in rat plasma was 0.018 ± 0.012 μg/mL and 0.132 ± 0.137 μg/mL for dosages of 1 g/kg and 3 g/kg, respectively. Geniposide had a C_max_ of 0.145 ± 0.251 μg/mL at a dosage of 1 g/kg and a C_max_ of 0.604 ± 0.256 μg/mL at the higher dosage. The observed C_max_ for rhein in the group receiving 3 g/kg was 1.659 ± 0.805 μg/mL, which was significantly increased compared to 0.311 ± 0.166 μg/mL in the 1 g/kg group. Although the significant difference in maximum concentration between the two dose groups, the time to reach the maximum concentration showed no significant differences for the three bioactive components. The results indicated that the absorption of scoparone and rhein was rapid (less than half an hour) and the increased dosage also altered the absorption of these three bioactive compounds (Table 5).

In addition, the areas under the plasma concentration-time curve (AUCs) of scoparone, geniposide and rhein were, respectively, 7.123 ± 3.379 min μg/mL, 94.88 ± 32.28 min μg/mL and 198.5 ± 51.3 min μg/mL in the 3 g/kg group, which were significantly increased 7.5-fold, 5.1-fold and 6.8-fold, respectively, compared to the group of 1 g/kg (*p*-value < 0.01). If the AUC was adjusted to the administrating dose, the high-dose group still had ratios that were 2.5 times, 1.7 times and 2.3 times higher for scoparone, geniposide and rhein, respectively, than for the low-dose group. All three compounds revealed a significant decrease in total body clearance (CL) with increased dose. The increase in the dose of the compounds resulted in a decrease in clearance and a more than proportional increase in drug AUC, implying saturation of the compounds or that more kinetic processes were not through simple first-order kinetics.

## 3. Discussion

Scoparone is one of the main active and important constituents of *Artemisia capillaris* Thunb (Yin-Chen-Hao). It has attracted attention and generated interest from researchers for further investigation due to prevention and treatment of liver disease [34,35]. Geniposide is well-known and identified in nearly 40 plants. It has been pharmacologically shown to have biological activities, including anti-inflammatory, antioxidative, anti-diabetic, neuroprotective, hepatoprotective and cholagogic effects. Similar properties, such as antitumor [36], anti-inflammation, antibacterial [37], anti-oxidation [38], antifibrosis and scavenging free radicals [39] were also discovered for rhein. Scoparone, geniposide and rhein are considered the main bioactive compounds of Yin-Chen-Hao-Tang and their synergistic effects contribute to the therapeutic purpose of YCHT reported for treating liver fibrosis [40], primary biliary cirrhosis [41], cholestasis, hepatitis [35], or pancreatic carcinoma [42]. Despite the crucial properties, seldom articles have simultaneously investigated these three bioactive compounds of YCHT. This study was the first attempt to address the pharmacokinetic characteristics of the bioactive compounds in YCHT based on doses proportionally administered orally in rats.

Our pharmacokinetic study results showed the bioactive compounds of scoparone, geniposide and rhein after administration of Yin-Chen-Hao-Tang (YCHT) at common therapeutic doses. Consistent with the previous pharmacokinetic study of YCHT [30], the times to reach the maximum concentrations of scoparone and rhein in rat plasma were less than one hour, which indicates rapid absorption of bioactive compounds. Furthermore, rats administered the higher dose had no disparity in time to reach the maximum concentration, with a proportionally greater maximum concentration than that of the low dose. The pharmacokinetic results showed an extensive capacity for absorption of scoparone, geniposide and rhein with this formula.

The AUCs of scoparone, geniposide and rhein in the formula at low and high doses were increased 7.5-fold, 5.1-fold and 6.8-fold, respectively. If we adjusted AUC by dose, it was, respectively, 2.5 times, 1.7 times and 2.3 times greater in the high-dose group than in the low-dose group. The clearance of these three compounds also decreased as the dose increased. The increase in dose was not proportional to the increase in AUC, with the change in clearance showing nonlinear PK properties of the bioactive compounds in the YCHT formula in the therapeutic range used to treat patients. Regarding the principles of pharmacokinetics, nonlinearity may be encountered at different kinetic levels of absorption, distribution, metabolism, or elimination. Nonlinearity at absorption is characterized as a less than proportional increase in the drug reaching systemic circulation as dose increases [43]. This phenomenon increases from saturation of a carrier system, protein binding, or gastrointestinal blood flow or mobility that are not in correspondence with our pharmacokinetics results, in which plasma concentration was elevated as the dose increased.

Nonlinearity at levels of metabolism or elimination is commonly presented and is saturable for metabolism due to drug-enzyme intermediates that are further processed to produce metabolites and recycled enzymes. Otherwise, increased AUC/dose ratio and elevated high plasma concentrations can also result from the saturation of renal tubular secretion that may saturate plasma protein binding, leading to an increase in the free compound and glomerular filtration or hepatic clearance [44]. The overall progress produces capacity-limited metabolism and should follow Michaelis-Menten kinetics [45]. However, few studies have demonstrated pharmacokinetic parameters in the dose relation of these candidate compounds, such as scoparone, geniposide and rhein. The clue to nonlinear pharmacokinetics may depend on the metabolism of the compounds. Scoparone had been presented at the highest level of distribution in the liver and decreased rapidly in the kidney, which supported that the primary metabolic pathway was via the microsomal cytochrome P450 enzyme [46]. Previous research reported that only CYP1A2 produced evident metabolites of scoparone even though several CYPS had been tested [47] and the major metabolic pathway of scoparone is demethylation in humans and several animal species [48,49]. Similar to scoparone, geniposide was found not to be hydrolyzed to genipin by rat liver homogenate hydrolyzed aryl β-d-glucoside or subcellular fractions such as lysosomes or microsomes and only one β-d-glucosidases from Eubacterium A-44 could transform geniposide in humans [50]. In addition, a previous study reported that the metabolism of rhein was mainly through hydroxylation depending on the presence of NADPH, which was mainly P450 2C9 and conjugation with glutathione only in the presence of cytosolic glutathione S-transferases. The above single and major metabolic pathways of the bioactive compounds may lead to the nonlinear pharmacokinetics found in our study.

Another phenomenon that could lead to nonlinear kinetics may lie in the synergetic interaction in the herbal formula. The synergistic effect was proposed based on the increased AUC in the equivalent bioactive components. For instance, oral administration of geniposide, Gardenia fruits and Gardenia herbal formulation produced better bioavailability than the pure compound [51]. A former experiment that measured rhein in the pure compound, single herb *Rheum palmatum* L. and Chinese herbal formula revealed an elevated AUC in the herbal formula [33]. Linear pharmacokinetics were found after oral administration of 50–200 mg of rhein in healthy Chinese subjects [52]. Furthermore, the pharmacokinetic parameters including bioavailability of scoparone or geniposide were found to be diversified along with formula composition changed [53]. These studies proposed that a potential pharmacokinetic interaction could be elicited between herbal ingredient–ingredient or herb-herb interactions. The impact on the contribution of the synergistic, additive, or antagonistic effects of an herbal formula to the pharmacokinetics seemed beyond prediction from a single or pure compound. As a consequence, increasing doses of the herbal formula may alter the pharmacokinetics due to potential interactions. Our experimental results are in agreement with previous studies and further complement the above evidence that possible accumulation of bioactive compounds in YCHT could occur in pharmacokinetics.

## 4. Materials and Methods

### 4.1. Materials and Reagents

Reference standards of scoparone (6,7-dimethoxycoumarin) was purchased from Sigma-Aldrich Research Biochemicals Inc. (St. Louis, MO, USA), geniposide was provided by Nacalai Tesque (Kyoto, Japan) and rhein was from Sigma-Aldrich, too. Carbamazepine was procured from Research Biochemicals International Inc. (Natick, MA, USA). Methanol (MeOH) was LC/MS grade and same as ammonium acetate and formic acid (98–100%), were all bought from E. Merck (Darmstadt, Germany). A Q-Gard 1 Purification Cartridge water purification system (Millipore, Bedford, MA, USA) coupled with a Millipak 40 Gamma Gold filter (0.22 µm) was used to produce triply deionized water. Reagents were analytical grade if not otherwise stated.

### 4.2. Liquid Chromatography and Tandem Mass Spectrometry

The UPLC system consisted of a CBM-20A system controller, LC-20AD XR pumps, a DGU-20A3 degasser, an SIL-20AC XR autosampler and a CTO-20A column oven coupled with an electrospray ionization (ESI) interface equipped LCMS-8030 triple quadrupole mass spectrometer (Shimadzu, Kyoto, Japan). The mass spectrometer was operated using multiple reaction monitoring (MRM) in positive or negative ion mode and argon gas was used for collision-induced dissociation (CID). The operating condition was optimized at a set interface voltage of 3.5 kV, a desolvation line temperature of 250 °C and the DL voltage (V) was scan, a heat block temperature of 400 °C and a collision gas pressure of 230 kPa; the nebulizing gas was N_2_ with flow of 0.5 L/min. The desolvation gas and drying gas were nitrogen and the gas flow rate was 3 L/min and 17 L/min.

All samples were subjected to chromatographic separation using the Shimadzu UPLC system with an Acquity UPLC BEH C18 column (1.7 µm, 100 mm × 2.1 mm) (Waters Corp., Milford, MA, USA). Samples were maintained in the autosampler at 4 °C with 10 µL of each being injected into the column. Chromatographic separations were conducted at 35 °C with a flow rate of 0.2 mL/min. The mobile phase was 1 mM ammonium acetate with 0.1% formic acid and methanol. A gradient elution was used from 20% methanol (0–2 min) to 95% methanol (3–11 min) then decreased to 20% methanol at 12 min. The overall run time is 20 mins. MRM transitions were scoparone (*m*/*z* 207 → 151), geniposide (*m*/*z* 406 → 227), rhein (*m*/*z* 283 → 240) and carbamazepine (*m*/*z* 237 → 194) for quantitation. The used specific ion mass and collision energy for quantitation are shown in the table (Table 1). Data acquisition was controlled with LabSolutions v. 5.60 SP1 software (Shimadzu, Kyoto, Japan).

### 4.3. Working Solutions and Quality Control (QC) Samples 

The stock of standard solution was prepared as scoparone, geniposide and rhein in methanol at a concentration of 1 mg/mL. Calibration standards were prepared by spiking appropriate amounts of the standard solution in drug-free rat plasma samples to yield final concentrations of 5, 10, 50, 100, 500 and 1000 ng/mL for each bioactive compound. All standard solutions were stored at −20 °C in polypropylene tubes when not in use. QC samples containing these three compounds were prepared in the same manner. 

### 4.4. Sample Preparations 

Sample preparations were made using the protein precipitation process. A forty-five-microliter aliquot of blank plasma sample was mixed with 5 µL of internal standard solution and 100 μL acetyl nitrate was added. The mixture was vortexed for 5 min and centrifuged at 16,000× *g* for 10 min at 4 °C. The supernatant layer was then collected and transferred through a 0.22 μm membrane filter (0.22 μm, Millex-GV, Millipore, USA) to 1.5 mL autosampler vials. Finally, 20 µL was used for injection into the UPLC-MS/MS system. 

### 4.5. Determination of Bioactive Contents from the Pharmaceutical Product

Pharmaceutical products of Yin-Chen-Hao-Tang (YCHT) were weighed and diluted to a concentration of 100 mg/mL. Samples were taken to the ultrasonic water bath for 10 min at room temperature followed by centrifugation at 13,000 rpm for 10 min at 4 °C. The supernatant then through a 0.22 μm filter and was ready to be sent to LC-MS/MS for analysis. The liquid chromatography conditions were same as mentioned above. Samples were identified in triplicate and the intra- and inter-day precision (% RSD) and accuracy (% bias) values of analytes were within 15%, guaranteeing that the analysis method provides excellent quantitation. The contents of the bioactive components were found to be 0.207 ± 0.006 mg/g for scoparone, 7.241 ± 0.139 mg/g for geniposide and 0.093 ± 0.002 mg/g for rhein.

### 4.6. Method Validations

All works were carried out through complete method validation based on the industrial guidelines for bioanalytical method validation from the US FDA [54].

#### 4.6.1. Specificity, Selectivity, LLOQ and Linearity

The blank rat plasma samples from six sources, blank plasma samples spiked with scoparone, geniposide and rhein at the lower limit of quantification (LLOQ) and plasma samples obtained from PK studies was analyzed to ascertain the specificity and selectivity of the method for endogenous plasma matrix components. The limits of detection (LOD) and lower limits of quantitation (LLOQ) were determined, respectively, as signal-to-noise ratios (S/N) of 3 and 10 for each standard in the optimized conditions. Five calibration curves containing at least six QC and non-zero concentrations were analyzed to generate the linearity of the developed method. Each calibration curve had to meet its acceptance criteria of simple linear regression of an r^2^ greater than 0.995, except LLOQ, which is accepted within 20% of nominal concentration. Otherwise, all the values needed to be within 15%.

#### 4.6.2. Accuracy and Precision

The inter-day precision and accuracy were evaluated by assaying five different QC samples on the same day and intra-day values were processed over three consecutive days. The precision was calculated as the relative standard deviation (RSD) from the observed concentration: precision (RSD, %) = [standard deviation (SD)/C_obs_] × 100%. The accuracy was calculated as the deviation of estimated concentration and nominal concentrations: accuracy (bias, %) = [(C_nom_ − C_obs_)/C_nom_] × 100%. The inter-day and intra-day accuracy and precision value have to be within ±15% of nominal values to meet acceptable reproducibility concentrations.

#### 4.6.3. Extraction Recovery and Matrix Effect

The extraction recoveries and matrix effects of scoparone, geniposide and rhein were evaluated to investigate the efficiency of the assay process. Recovery was calculated comparing the mean peak area of the analytes spiked before extraction (R3) to the mean peak area of analytes spiked post-extraction (R2) in three representative low, middle and high concentrations (10, 100 and 1000 ng/mL). Matrix effects are also determined at the same three QC concentrations and were calculated as the ratio of the analytes spiked post-extraction (R2) to the same analytes in neat standard solution (R1). Recovery should be consistent, precise and reproducible as per acceptance criteria. The matrix effect values are considered ionization suppression if less than 85% and ionization enhancement if more than 115%.

### 4.7. Application of the Method to a Pharmacokinetic Study

Male Sprague Dawley rats weighing 230 ± 30 g were used for the following pharmacokinetic (PK) study. The rats were housed under controlled environmental conditions with free access to food and drinking water up to 12 h prior to experiments. The experimental protocol was approved by the Institutional Animal Ethics Committee of National Yang-Ming University, Taipei, Taiwan (IACUC1070112). All rats divided into two experimental groups were intragastrically fed pharmaceutical herbal product formula Yin-Chen-Hao Tang suspended in normal saline at 1 g/kg or 3 g/kg corresponding to the clinical daily dose of 9.7 g and 29.2 g in a 60 kg adult [55]. Blood samples were collected via a polyethylene tubing (PE-50) implanted priory in the jugular vein of each rat in a heparin-rinsed vial. An aliquot of 100–120 µL blood was collected at time intervals of 0, 5, 15, 30, 60, 90, 120, 180, 240, 300 and 360 min after oral administration of the herbal product. Each group contained a total of six rats to diminish individual variation. All blood samples were immediately centrifuged as mentioned above (16,000× *g* for 10 min at 4 °C) and stored at −20 °C until analysis.

### 4.8. PK Parameters and Statistical Analysis

The pharmacokinetic parameters were calculated using the pharmacokinetic software WinNonlin Standard Edition, version 1.1 (Scientific Consulting Inc., Apex, NC, USA). The area under the plasma concentration-time curve (AUC), terminal elimination half-life (t_1/2_) and oral clearance (CL) were estimated using non-compartmental analysis. The maximum plasma concentration (C_max_) and time to reach the maximum concentration (T_max_) was directly applied from the plasma concentration-time curve. Pharmacokinetic parameters of each individual data set were calculated using WinNonlin Standard Edition, version 1.1 (Pharsight Corp., Mountain View, CA, USA). Statistics and graphics were performed with SPSS version 18.0 (SPSS Inc., Chicago, IL, USA) and SigmaPlot 10.0 software (Systat Software Inc., San Jose, CA, USA). All values were expressed as the mean ± standard deviation (S.D.).

## 5. Conclusions

An UPLC-MS/MS method was developed in rat plasma to determine the bioactive compounds in a common traditional herbal formula used for chronic liver disease. The assay had good sensitivity and specificity for simultaneous determination of scoparone, geniposide and rhein in rat plasma and achieved good accuracy and precision for bioanalytical method validation. The pharmacokinetic features of scoparone and rhein were exhibited a rapid oral absorption following oral administration of YCHT and a beyond proportional AUC and C_max_ of all these bioactive compounds was discovered as the administered dose increased. This study is the first study to demonstrate the pharmacokinetics of scoparone, geniposide and rhein simultaneously in two doses. These PK data may not only help to elucidate the metabolic mechanism of YCHT formulation but may also help to design effective ways to apply this formula at a high dose.

## Figures and Tables

**Table 1:** The LC-MS/MS analytical conditions for the identification of the bioactive constituents.

Constituents	Molecular Weight	RT ^1^ (min)	Selected *m*/*z*		Collision Energy (eV)
			Q1 *m*/*z*	Q3 *m*/*z*	
Scoparone	206.57	5.3	207	151	−22
Geniposide	388.14	4.7	406	227	−10
Rhein	284.03	6.9	283	239	14
Carbamazepine	236.10	5.8	237	194	−20

^1^ RT: retention time.

**Table 2:** Calibration curve of scoparone, geniposide and rhein.

Constituents	Calibration Curve	Range (ng/mL)	R^2^	LOD (ng/mL)
Scoparone	y = 0.0195x − 0.0136	5–500	0.9999	1
Geniposide	y = 0.0075x − 0.0066	10–1000	0.9998	5
Rhein	y = 0.007x − 0.001	10–1000	0.9999	5

**Table 3:** Intra-day and inter-day precision and accuracy of scoparone, geniposide and rhein in plasma samples.

Nominal Concentration (ng/mL)	Observed Concentration (ng/mL) ^1^	Precision (%)	Accuracy (%)	Observed Concentration (ng/mL) ^1^	Precision (%)	Accuracy (%)
	Intra-Day			Inter-Day		
Scoparone						
5	5.2 ± 0.1	2.6	3.4	5.1 ± 0.1	1.7	2.0
10	10.6 ± 0.8	7.9	5.6	10.0 ± 0.4	4.1	0.2
50	49.9 ± 1.2	2.4	−0.2	48.1 ± 1.3	2.7	−3.7
100	99.9 ± 0.7	0.7	−0.1	100.4 ± 3.0	3.0	0.4
500	500.3 ± 0.4	0.1	0.1	499.4 ± 8.3	1.7	−0.1
Geniposide						
10	10.7 ± 0.7	6.9	7.2	10.7 ± 0.4	3.6	6.6
50	49.7 ± 0.9	1.8	−0.7	50.6 ± 2.5	5.0	1.2
100	99.6 ± 1.4	1.4	−0.5	100.0 ± 3.0	3.0	−0.1
500	500.7 ± 1.5	0.3	0.1	505.2 ± 5.9	1.2	5.2
1000	999.3 ± 1.8	0.2	−0.1	1014.1 ± 31.6	3.1	1.4
Rhein						
10	9.8 ± 0.7	6.8	−2.3	9.8 ± 0.2	1.6	−1.7
50	48.8 ± 0.7	1.4	−2.4	49.4 ± 0.9	1.7	−1.1
100	100.0 ± 2.8	2.8	0.0	97.0 ± 1.7	1.8	−3.0
500	502.4 ± 2.7	0.5	0.5	489.2 ± 10.6	2.2	−2.2
1000	998.5 ± 2.5	0.3	−0.2	988.1 ± 30.4	3.1	−1.2

^1^ Data are expressed as the means ± S.D. (*n* = 6). Precision: RSD (%) = (standard deviation/C_obs_) × 100; Accuracy: Bias (%) = [(C_obs_ − C_nom_)/C_nom_] × 100.

**Table 4:** Extraction recovery and matrix effect of the marker constituents.

Nominal Conc. (ng/mL)	R 1	R 2	R 3	ME (%) ^1^	RE (%) ^2^
Scoparone					
10	44,912 ± 1152	48,721 ± 2536	41,883 ± 1236	113.0 ± 16.7	99.8 ± 13.1
100	427,813 ± 14,929	478,693 ± 2688	424,339 ± 15,638	109.5 ± 2.0	99.5 ± 3.5
1000	4,400,014 ± 56,880	4,332,780 ± 178,478	3,636,719 ± 220,707	97.5 ± 1.5	99.7 ± 5.2
mean ± SD				106.7 ± 6.7	99.7 ± 7.2
Geniposide					
10	4690 ± 482	7474 ± 582	5863 ± 412	167.7 ± 34.5	91.2 ± 10.6
100	49,536 ± 2178	67,386 ± 1831	54,524 ± 2940	133.5 ± 11.3	90.9 ± 7.1
1000	649,073 ± 7233	771,802 ± 15,246	607,190 ± 24,396	117.9 ± 5.9	93.5 ± 3.5
mean ± SD				139.7 ± 17.2	91.9 ± 7.1
Rhein					
50	15,161 ± 597	12,484 ± 309	12,858 ± 653	85.1 ± 5.2	121.5 ± 11.1
100	27,608 ± 879	24,708 ± 1230	23,412 ± 1000	87.6 ± 3.7	106.5 ± 5.8
1000	330,092 ± 2490	285,450 ± 3193	241,434 ± 977	85.8 ± 5.1	100.5 ± 2.0
mean ± SD				86.2 ± 4.7	109.5 ± 6.3
Carbamazepine (IS)					
10	289,244 ± 6018	282,335 ± 3741	244,125 ± 3117	99.9 ± 2.9	85.7 ± 5.2

Data are expressed as the mean ± S.D. (*n* = 3). ^1^ ME: Matrix effect (%) calculated as (R 2/R 1) × 100%. ^2^ RE: Recovery (%) calculated as (R 3/R 2) × 100%.

**Table 5:** Pharmacokinetic parameters of scoparone, geniposide and rhein in rat plasma after administration of the herbal formula Yin-Chen-Hao-Tang.

Parameters	YCHT 1 g/kg			YCHT 3 g/kg		
	Scoparone	Geniposide	Rhein	Scoparone	Geniposide	Rhein
C_max_ (μg/mL)	0.018 ± 0.012	0.145 ± 0.251	0.311 ± 0.166	0.132 ± 0.137 *	0.604 ± 0.256 **	1.659 ± 0.805 **
T_max_ (min)	14 ± 20	81 ± 71	12 ± 5	21 ± 20	81 ± 77	23 ± 19
AUC (min μg/mL)	0.955 ± 1.168	18.76 ± 28.45	29.11 ± 13.13	7.123 ± 3.379 **	94.88 ± 32.28 **	198.5 ± 51.3 **
AUC/dose	-	-	-	2.374 ± 1.126	31.63 ± 10.76	66.18 ± 17.09
t_1/2_ (min)	26 ± 5	80 ± 18	121 ± 46	69 ± 55	86 ± 29	104 ± 69
CL (mL/min/kg)	394.7 ± 209.4	1403 ± 1018	3.710 ± 1.366	131.5 ± 123.6 *	259.8 ± 109.2 *	1.483 ± 0.357 **
MRT (min)	19 ± 14	115 ± 31	106 ± 12	51 ± 18 **	152 ± 20 *	116 ± 20

Data are expressed as the mean ± S.D. (n = 6). *T*-test was significant: * *p* < 0.05; ** *p* < 0.01. YCHT, Chinese herbal formula Yin-Chen-Hao-Tang; C_max_ = Maximum concentration; T_max_ = Time of maximum concentration; AUC = Area under the curve; t_1/2_ = Elimination half-life; CL = clearance; MRT = mean retention time.
